# Role of Glucocorticoids in Metabolic Dysfunction-Associated Steatotic Liver Disease

**DOI:** 10.1007/s13679-024-00556-1

**Published:** 2024-03-08

**Authors:** Stergios A. Polyzos, Giovanni Targher

**Affiliations:** 1 First Laboratory of Pharmacology, School of Medicine, Aristotle University of Thessaloniki, Thessaloniki, 54124 Greece; 2 Department of Medicine, University of Verona, Verona, Italy; 3 Metabolic Diseases Research Unit, IRCCS Sacro Cuore - Don Calabria Hospital, Negrar di Valpolicella (VR), Italy

**Keywords:** Adrenals, Cortisol, Glucocorticoids, Metabolic dysfunction-associated steatotic liver disease, Metabolic dysfunction-associated steatohepatitis, Nonalcoholic fatty liver disease

## Abstract

**Purpose of the Review:**

To summarize published data on the association between glucocorticoids and metabolic dysfunction-associated steatotic liver disease (MASLD), focusing on the possible pathophysiological links and related treatment considerations.

**Recent Findings:**

Glucocorticoids, commonly used for managing many inflammatory and autoimmune diseases, may contribute to the development and progression of MASLD. Glucocorticoids may induce hyperglycemia and hyperinsulinemia, thus increasing systemic and hepatic insulin resistance, a hallmark of MASLD pathogenesis. Furthermore, glucocorticoids increase adipose tissue lipolysis, and hepatic *de novo* lipogenesis and decrease hepatic fatty acid β-oxidation, thus promoting MASLD development. Preclinical evidence also suggests that glucocorticoids may adversely affect hepatic inflammation and fibrosis. 11beta-hydroxysteroid dehydrogenase type 1 (11β-HSD1) and 5α-reductase are implicated in the link between glucocorticoids and MASLD, the former enzyme increasing and the latter reducing the glucocorticoid action on the liver. Treatment considerations exist due to the pathogenic link between glucocorticoids and MASLD. Since iatrogenic hypercortisolism is common, glucocorticoids should be used at the minimum daily dose to control the subjective disease. Furthermore, the pharmacologic inhibition of 11β-HSD1 has provided favorable results in MASLD, both in preclinical studies and early MASH clinical trials.

**Summary:**

Glucocorticoids are closely linked to MASLD pathophysiology, with specific clinical and therapeutic implications.

## Introduction

Metabolic dysfunction-associated steatotic liver disease (MASLD), previously known as nonalcoholic fatty liver disease (NAFLD) [1••], is a highly prevalent condition worldwide (affecting up to ~30% of the general adult population) without any approved pharmaceutical treatment [2]. The raising global prevalence of MASLD parallels the increase in obesity worldwide [3]. MASLD prevalence rises up to ~90% in severely obese individuals [4]. Notably, obesity does not only increase the risk of developing MASLD, but also the progression to more advanced forms of liver disease, such as metabolic dysfunction-associated steatohepatitis (MASH) and cirrhosis [5]. The close association between obesity and MASLD is reflected in the newly proposed diagnostic criteria of the disease [1••]: obesity is one of the five common cardiometabolic criteria used for diagnosing MASLD. Specifically, the presence of obesity alone in a patient with hepatic steatosis (identified by imaging methods or liver biopsy) establishes a diagnosis of MASLD [1••]. Although obesity is an important risk factor for MASLD, the pathogenesis of this liver disease is complex and multifactorial. According to the so-called “multiple-hit” hypothesis, several pathogenic contributors (“hits”) act in parallel and for different periods in the pathogenesis of MASLD in each affected individual [6]. The embracement of this pathogenetic “multiple-hit” model may have important therapeutic implications since different pathogenic contributors should be managed in each patient with NAFLD/MASLD [7]. This may also partly account for the failure of most recent MASLD clinical trials because *“one pill does not seem to fit all”*, so an individualized combination treatment for NAFLD/MASLD seems to be more suitable [7].

Glucocorticoids (GC) have been early proposed as one of the multiple contributing factors (“hits”) predisposing not only to MASLD, but also to more advanced forms of the disease, including nonalcoholic steatohepatitis (NASH) [8], recently renamed to MASH [1••]. Notably, NASH/MASH is not regarded as a common histological feature of drug effect, although it has been estimated that about 2% of NASH/MASH cases may be drug-induced, GC are included among the medications related to it [9]. GC may adversely affect the liver mostly indirectly by affecting insulin resistance (IR), glucose and lipid metabolism [8], which are all mechanisms potentially implicated in the pathogenesis of NAFLD/MASLD [10]. It is also highlighted that NAFLD/MASLD is a common manifestation of Cushing’s syndrome, a condition characterized by chronic hypercortisolism, i.e., GC excess [11]. However, it is difficult to differentiate to what extent the excess of GC causes MASLD or drug-induced liver injury (DILI). Although controversy still exists, it has been suggested that DILI refers to a direct effect of a drug on the liver cells [9]. In contrast, MASLD refers to the indirect effects of a drug on mechanisms contributing to the pathogenesis of hepatic steatosis, including but not limited to IR, altered glucose and lipid metabolism, and mitochondrial dysfunction [11]. The newly proposed change in fatty liver disease terminology, accompanied by a change in the definition of the disease, simplifies this terminology-related controversy, because both MASLD and DILI are now included under the umbrella of steatotic liver disease (SLD) [1••]. Apart from the rationale that the results of a drug’s hepatic effect are the same, regardless of the term MASLD or DILI, the new terminology may facilitate the investigation of mixed types of SLD, e.g., those with MASLD and DILI [12], e.g., patients with obesity, type 2 diabetes mellitus (T2DM) or dyslipidemia who are on GC treatment, since DILI is not excluded [1••], as in the case of NAFLD. For such reasons, the recently proposed new terminology of MASLD and SLD [1••] is hereby selected.

This narrative review aimed to summarize published data on the association between GC and SLD, focusing on the possible pathophysiological links and related treatment considerations.

## Literature Search

We performed a systematic literature search in PubMed, not limited by publication time. By combining Medical Subject Heading (MeSH) terms and non-MeSH terms, the following string was developed, which was the main body of our electronic search: (("Glucocorticoids"[Mesh]) OR ("Cushing Syndrome"[Mesh]) OR (Cushing's syndrome) OR (Cushing's disease) OR (Cushing disease) OR (Cushing syndrome) OR (glucocorticoids)) AND (("Non-alcoholic Fatty Liver Disease"[Mesh]) OR (nonalcoholic fatty liver disease) OR (nonalcoholic steatohepatitis) OR NASH OR NAFLD OR MAFLD OR (Metabolic dysfunction associated fatty liver disease) OR (Metabolic associated fatty liver disease) OR (Metabolic dysfunction associated steatotic liver disease) OR MASLD OR MASH OR (steatotic liver disease)). Following this search string, 298 articles were retrieved (last update: February 10, 2024).

Since this is a narrative review, some articles outside the results of this search were added at the authors' discretion, when this was deemed necessary for the review.

## Pathophysiology

GC are synthesized and secreted from the adrenal cortex (zona fasciculata) under the control of the hypothalamus-pituitary-adrenal (HPA) axis; cortisol is the most important GC produced in humans, and corticosterone is the most important GC produced in rodents [13]. GC are physiologically implicated in the regulation of carbohydrate, lipid and protein metabolism, as well as energy homeostasis, but also have multiple and diverse actions, including, but not limited to, growth, reproduction and immune response [13, 14]. Given their multi-faceted actions, excess GC has been linked to multisystem morbidity, including abdominal obesity, T2DM, atherogenic dyslipidemia and hypertension, which may predispose to the development of cardiovascular disease (CVD), such as left ventricular dysfunction/hypertrophy, ischemic heart disease and heart failure [15]. It should be highlighted that abdominal obesity, T2DM, atherogenic dyslipidemia and hypertension are also closely associated with MASLD, thus further increasing the risk of CVD, which represents the leading cause of death in people with MASLD [16••]. Therefore, it seems that GC can impact the pathogenesis of MASLD, possibly through multiple mechanisms, including, but not limited to, dysregulation of glucose and lipid metabolism. As a result of these GC actions on the liver, corticosterone-treated mice had greater liver weight and hepatic steatosis (together with expanded adipose tissue), increased IR, and higher serum glucose, triglycerides and alanine aminotransferase (ALT) concentrations than control mice [17]. As expected, the weight of the adrenal glands of these corticosterone-treated mice was reduced compared with that of control mice, owing to the HPA axis suppression [17].

The biological effects of GC are mainly achieved via activation of the nuclear glucocorticoid receptor (GR) [18]. More specifically, the knock-down of hepatic GR in mouse models improved hepatic steatosis; GR may act partly by repressing the transcription of the *hairy enhancer of the split 1* (*Hes1*) gene [18]. HES1 is a master transcription factor whose GC-dependent silencing is necessary for GR to regulate many target genes [19]. It should be noted that GC, through their specific GR, may regulate about 25% of the genome [19]; therefore, the GC effects on the liver, which are the focus of this review article, may constitute only a part of the wide range of GC actions in diverse organs and tissues, which probably cross-talk each other. Besides the GR as the primary receptor mediating GC actions, the mineralocorticoid receptor (MR) may also bind GC and mediate a part of the hepatic actions, despite our limited knowledge on the topic and conflicting to-date results. Notably, it has been shown that a high-fat diet (HFD) in mice with adipocyte-specific deletion of MR led to reductions in body weight, fat mass and hepatic steatosis compared to control mice [20•]; of note, decreased expression of genes associated with adipogenesis in the white adipose tissue of MR knock-out mice was also shown, thus implying that MR may mediate adipogenesis, hence affecting the liver [20•].

Below, the main molecular mechanisms possibly linking GC and MASLD are discussed.

### Effects of GC Excess on IR and Glucose Metabolism

IR plays a pathogenic role in MASLD development [10] and represents an important mechanism through which GC may adversely affect MASLD. It was early shown that patients with active Cushing’s syndrome have markedly increased systemic IR [21]. GC-induced IR is closely related to both the liver and skeletal muscle.

In the liver, GC may decrease the phosphorylation of insulin receptor substrate (IRS)-1 and phosphatidylinositol-3-kinase (PI3K), thus reducing the actions of key intracellular molecules of the insulin signaling pathway [22]. Furthermore, GC may stimulate gene transcription of key molecules of hepatic gluconeogenesis, e.g., the glucose-6-phosphatase catalytic subunit (G6Pase), which contributes to the final steps of gluconeogenesis, thus increasing hepatic glucose production [23]. Moreover, GC may also mediate the effects of glucagon on the liver, i.e., potentiating gluconeogenesis [24]. Hepatic gluconeogenesis seems to be facilitated by GC-induced protein degradation, which provides amino acids, i.e., substrates for gluconeogenesis [13]. The above considering, GC may impair hepatic insulin signaling and increase hepatic glucose production, two processes that may cross-talk each other and lead to dysregulation of glucose metabolism and MASLD. Notably, GC may also increase glycogen synthesis in the liver by activating glycogen synthase and inactivating glycogen phosphorylase [25]. This mechanism is considered counteracting, i.e., restoring the ability to synthesize and accumulate hepatic glycogen after stress [26].

In the skeletal muscle, GC may decrease glucose uptake [21], leading to hyperinsulinemia as a counteracting mechanism to surpass hyperglycemia. A direct effect of GC on post-receptor insulin signaling was also shown in the skeletal muscle: specifically, the expressions of IRS-1 and PI3K were decreased, as well as PI3K phosphorylation, thus reducing the translocation of glucose transporter (GLUT)-4 on the cell membrane; consequently, glucose uptake by the skeletal muscle was reduced [22, 27]. An indirect effect of GC has also been supported, principally based on the impact of GC on protein and lipid metabolism; specifically, GC may decrease protein synthesis and increase protein degradation, thus increasing amino acids, which may further impair intracellular insulin signaling [13]. GC may also increase peripheral lipolysis in the adipose tissue and, consequently, the increased levels of free fatty acids (FFAs) impair insulin signaling [13]. Furthermore, acute and short-term administration of GC was shown to impair pancreatic insulin secretion, thus further worsening hyperglycemia and IR [28]. This is partly attributed to the decreasing effect of GC on GLUT2 and glucokinase expressions, which are essential for glucose detection and stimulation of pancreatic insulin secretion, as shown in experimental studies [29, 30].

### Effects of GC Excess on Fatty Acid Metabolism

GC may play a crucial role in fatty acid (FA) and lipoprotein metabolism by acting in the adipose tissue and the liver. In the adipocytes, GC may increase peripheral lipolysis mainly by stimulating the gene expression of hormone-sensitive lipase (HSL) and adipose triglyceride lipase (ATGL) [31]. These GC-induced lipolytic effects are more prominent in subcutaneous adipose tissue (SAT) than in visceral adipose tissue (VAT) [32], and may be further amplified by the GC-induced IR [33]. Furthermore, GC may favor lipogenesis, by suppressing adenosine 5'-monophosphate-activated protein kinase (AMPK) activity, thus leading to lipid accumulation principally in VAT [34]; indeed, AMPK activity in VAT of patients with Cushing’s syndrome is markedly inhibited, thus partly explaining VAT expansion in these patients [35].

Because of increased lipolysis in the adipose tissue, circulating FFAs increase and are delivered to other tissues, such as the liver [31]. In the hepatocytes, GC may inhibit fatty acid β-oxidation and secretion of hepatic triglycerides (in the form of very-low-density lipoprotein [VLDL] particles) [36]; thus, lipolysis-derived FFAs are mostly converted to triglycerides and stored as lipid droplets in hepatocytes. GC may also promote hepatic *de novo* lipogenesis [37], i.e., increased production of FFAs from other substrates, e.g., fructose; thus, hepatic FFAs and triglyceride storage are further augmented. Overall, these GC-induced mechanisms can lead to hepatic steatosis (MASLD) development.

### Effects of 11beta-hydroxysteroid Dehydrogenase Type 1 activity

In humans, 11beta-hydroxysteroid dehydrogenase type 1 (11β-HSD1) is regarded as a key regulatory enzyme of cortisol action at the cellular level since it converts the inactive cortisone to active cortisol [38]. 11β-HSD1 over-expression in the adipose tissue of transgenic nonobese mice results in dyslipidemia and hepatic steatosis [39], whereas 11β-HSD1 over-expression in the liver results in more severe IR and MASLD [40]; this implies that some GC-induced effects on MASLD are not mediated by obesity. In line with these experimental findings, other authors also showed that adipose-specific transgenic 11β-HSD1 mice fed with HFD developed more severe hepatic IR and steatosis than control mice, thus underlying the pathogenic effect of adipose tissue 11β-HSD1 activity on hepatic IR and MASLD [41]. *Vice versa*, 11β-HSD1 knock-out mice were protected from hepatic IR, steatosis and other manifestations of hypercortisolism [42]. More importantly, adipose-tissue-specific 11β-HSD1 knock-out mice were similarly protected from excess circulating FFAs and hepatic steatosis, whereas liver-specific 11β-HSD1 knock-out mice were not [42]. These experimental findings imply that the adipose tissue 11β-HSD1 activity plays a more important role in MASLD than the liver 11β-HSD1 activity.

However, other investigators reported that 11β-HSD1 overexpression in a transgenic hepatocyte-specific 11β-HSD1 mouse model activated GR and promoted glycoprotein 78 (gp78) stimulation, leading to increased hepatic *de novo* lipogenesis, mainly through upregulating multiple key regulatory proteins, including sterol regulatory element-binding protein-1 (SREBP)-1, fatty acid synthase (FAS), stearoyl-CoA desaturase-1 (SCD1), and acetyl-CoA carboxylase 1 (ACC1) [43].

Apart from the direct effects of adipose tissue hypercortisolism on glucose and lipid metabolism, as mentioned above, the 11β-HSD1 activity in VAT may also result in portal hypercortisolism, i.e., a condition more directly affecting the liver in the long term [44]. In line with these experimental findings, 11β-HSD1 mRNA expression was higher in the liver of patients with MASH than in control subjects [45].

### Effects of 5α- and 5β-reductase Activity

5α-reductase is another enzyme involved in cortisol metabolism, beyond its classic role in converting testosterone to its 5α-reduced form, dihydrotestosterone (DHT). 5α-reductase has two major isoforms, type 1 and type 2, which are expressed in hepatocytes and convert cortisol to 5α-dihydro-cortisol, thereby inactivating it [46]. Mice knock-out for 5α-reductase type 1, but not for type 2, fed with a HFD, developed greater hepatic steatosis than wild-type mice [47]. Similarly, other authors showed that mice knock-out for 5α-reductase type 1 developed IR and hepatic steatosis and were more susceptible to fibrogenic stimuli in the liver [48]. Experimentally, other authors showed that 5β-reductase knock-down in human hepatoma cell lines increased hepatocyte triglyceride accumulation and glycogen synthesis, mainly through increased *de novo* lipogenesis and decreased FA β-oxidation, thus fueling hepatic inflammation [49].

In humans, the activity of 5α-reductase, as implied by the concentrations of its urinary metabolites, was higher in patients with MASLD than in controls [45]; it was hypothesized that this might represent a counteracting mechanism to protect the liver from damage induced by exposure to high cortisol levels [45], but it remains to be proven in mechanistic studies. Furthermore, 5β-reductase expression was inversely associated with hepatic steatosis, inflammation, and fibrosis in the human livers of obese individuals [49].

The above considering, the enzymatic activities of 5α- and 5β-reductase may be implicated in the pathogenesis of MASLD; however, further mechanistic and clinical studies are required to better elucidate this interesting topic.

### Effects of GC on Hepatic Inflammation and Fibrosis

All the above findings support a long-term effect of GC on hepatic/systemic IR and hepatic steatosis, but cannot directly explain the potential impact of GC on more severe liver disease, e.g., NASH/MASH and liver fibrosis, the latter considered the strongest histological prognostic factor of adverse liver-related and extrahepatic outcomes in people with MASLD [50]. Of course, the maintenance of hepatic steatosis in the long term is a prerequisite to the future development of MASH and hepatic fibrosis [51]. However, this observation of the natural history of MASLD does not provide specific information on the potential mechanistic effects of GC on hepatic inflammation and fibrosis. To date, there is limited evidence on this topic, which is presented hereby.

Adenovirus-mediated overexpression of GR (subtype β) resulted, apart from the development of hepatic steatosis, in the increase in tumor-necrosis factor-α (TNF-α) and activation of its downstream signaling, nuclear factor-kappaB (NF-kB), in HFD-treated mice [52]. TNF-α plays a role in developing hepatic steatosis but mainly in the progression of the disease to hepatic inflammation and fibrosis, as it is reviewed in detail elsewhere [53]. Furthermore, in the same mouse model, overexpression of GR (subtype β) was shown to result in the shifting of macrophages to M1, an activated macrophage phenotype that is closely related to hepatic inflammation [52].

Furthermore, it was shown that male Wistar rats exposed to stress displayed high 11β-HSD1 activity and increased oxidative stress, hepatic inflammation and fibrosis [54]. More importantly, the genetic and pharmacologic inhibition of 11β-HSD1 was beneficial for hepatic inflammation and fibrosis both *in vitro* and in various mouse models [55]. Specifically, 11β-HSD1 knock-down was shown to reverse the activation of hepatic stellate cells (HSCs), the master cells driving hepatic fibrogenesis; notably, 11β-HSD1 knock-down renders HSCs less susceptible to the effect of transforming growth factor (TGF)-β1, regarded as a key factor to their activation [55]. Furthermore, the pharmacologic inhibition of 11β-HSD1 with J2H-1702 reduced the expression of genes related to NF-κΒ signaling, including that of TNF-α, thus implying an attenuating effect on hepatic inflammation, as well as genes related to collagen I and II and fibronectin, implying an attenuating effect on hepatic fibrogenesis [55]. In line with these experimental findings, other authors also reported a diminishing impact of 11β-HSD1 inhibition on TNF-α and interleukin-6 production [56], which is also associated with NASH/MASH [57].

**Fig. 1:**
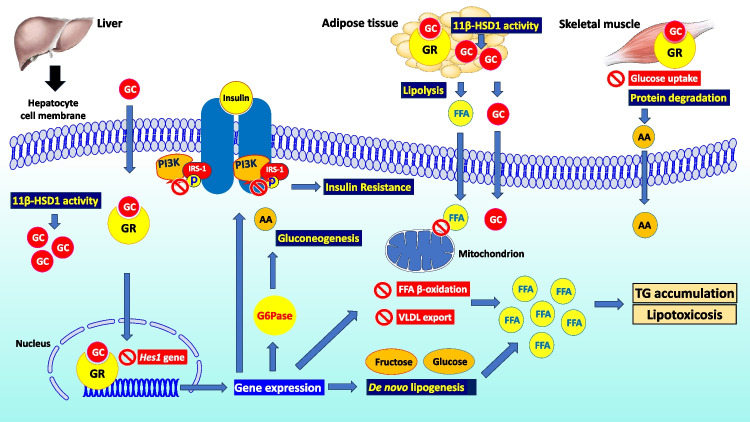
Pathophysiology of GC-induced MASLD. Endogenous and exogenous GC enter the hepatocytes, where they bind their specific GR and move into the nucleus, affecting the transcription of multiple genes. At the same time, GC may also repress the expression of the *Hes1* gene, which is regarded as a prerequisite for their action. GC action is enhanced by the activity of 11β-HSD1, which converts the inactive cortisone to active cortisol both in the hepatocytes and adipocytes. The primary actions of GC in the hepatocyte are: decrease in IRS-1 and PI3K phosphorylation, i.e., attenuating the action of key intracellular molecules of insulin signaling, thus inducing IR; IR is further impaired by the GC-induced decrease in glucose uptake in the skeletal muscle; increase in gene transcription of key intracellular molecules of hepatic gluconeogenesis, e.g., the G6Pase, which contributes to the final steps of gluconeogenesis; GC-induced protein degradation, e.g., in the skeletal muscle, provides amino acids as substrates for hepatic gluconeogenesis; decrease in fatty acid β-oxidation and in VLDL export from the hepatocytes, whereas simultaneously more FFAs enter the hepatocytes, owing to the GC-induced lipolysis in the adipose tissue; consequently, TG are accumulated into the hepatocytes and hepatic lipotoxicosis may occur; and increase in hepatic *de novo* lipogenesis, using carbohydrates as substrates, e.g., fructose or glucose, thus further increasing hepatic TG accumulation. Abbreviations: 11β-HSD1, 11beta-hydroxysteroid dehydrogenase type 1; AA, amino acid; FFA, free fatty acid; G6Pase, glucose-6-phosphatase catalytic subunit; GC, glucocorticoids; GR, glucocorticoid receptor; *Hes1*, *hairy enhancer of the split 1*; IR, insulin resistance; IRS, insulin receptor substrate; MASLD, metabolic dysfunction-associated steatotic liver disease; P, phosphoryl group; PI3K, phosphatidylinositol-3-kinase; TG, triglyceride; VLDL, very low-density lipoprotein

Therefore, despite limited evidence so far, GC excess adversely affects not only hepatic steatosis, but also hepatic inflammation and fibrosis; however, additional mechanistic and clinical studies are warranted on this important topic. Figure 1 schematically depicts key components of the complex interplay linking GC and MASLD.

## The Clinical Paradigm of Cushing’s Syndrome

Cushing’s syndrome is characterized by chronic hypercortisolism, thus being a typical paradigm for GC excess-induced clinical manifestations [15]. The causes of Cushing’s syndrome are usually classified into adrenocorticotropic hormone (ACTH)-dependent (e.g., Cushing’s disease, owing to an ACTH-secreting pituitary tumor) and ACTH-independent (e.g., a cortisol-secreting adrenal tumor) [15]. In clinical practice, iatrogenic (exogenous) Cushing’s syndrome, owing to the chronic pharmacological treatment of multiple and diverse inflammatory and autoimmune diseases with exogenous GC, is the commonest cause of Cushing’s syndrome and is included in the latter category [15]. In a registry of patients with non-exogenous Cushing’s syndrome (the European Registry on Cushing's syndrome [ERCUSYN]), high rates of weight gain (83%), arterial hypertension (79%) and T2DM (32%) were observed in patients with Cushing’s syndrome [58]; all these conditions are closely related to the diagnosis of MASLD [1••, 5, 59]. In a retrospective study in a Chinese population, similar rates of arterial hypertension (71%) and T2DM (39%) were observed, as well as high rates of atherogenic dyslipidemia (38%) [60], which is also related to the diagnosis of MASLD [1••, 61]. Of note, dyslipidemia observed in Cushing’s syndrome is mainly characterized by hypercholesterolemia, high triglycerides and low high-density lipoprotein cholesterol (HDL-C) levels [62].

Obesity and hepatic steatosis were observed in rats [63], mice [17], and other animals [64] with Cushing’s syndrome. Conversely, the first report of hepatic steatosis in a patient with Cushing’s syndrome was in 1983, three years after the first description of NASH [65]; in that early report, it was reported that Cushing’s syndrome is associated with unusual, i.e., extra-adipose tissue, fat deposition in ectopic sites, including the liver [66].

Some small clinical studies have examined the prevalence of MASLD in patients with Cushing’s syndrome, providing interesting results. In a UK study of 50 patients with active Cushing’s syndrome, hepatic steatosis (assessed by computerized tomography) was observed in 10 (20%) of these patients [67]. In this study, hepatic steatosis was also associated with greater total abdominal fat and visceral fat areas [67], thus further validating a close association between obesity and hepatic steatosis in patients with active Cushing’s syndrome. In a Chinese study of 73 patients with Cushing’s syndrome, the prevalence of hepatic steatosis on ultrasonography was over two times higher in men than in women (61.5% vs. 28.6%), possibly implying a gender-specific difference in MASLD prevalence [68]. However, it should be noted that the male and female patients of this study were relatively young (median age 30.0 and 33.5 years, respectively) [68], i.e., most women were premenopausal. Higher rates of MASLD in premenopausal women than in men of similar age were also observed in the general population, a difference that is attenuated after menopause when almost identical rates of MASLD are observed in both sexes [69]. In a case-control study of German patients with biochemically controlled Cushing's disease due to ACTH-secreting pituitary tumor (n = 33) and controls with non-functioning pituitary adenomas (n = 79), hepatic steatosis (non-invasively evaluated with the Fatty Liver Index [FLI] > 60) was present in 30.3% of patients with Cushing’s disease (non-treatment naïve), which was not statistically different compared with the prevalence of hepatic steatosis observed in those with non-functioning pituitary adenomas (20.3%). Importantly, in the same study, hepatic steatosis was also associated with hydrocortisone treatment (average daily dose) received by patients with adrenal insufficiency [70]. In another Chinese study, the rate of hepatic steatosis in young patients with Cushing’s syndrome (mean age 38.1 years) was 24.8% [60]. However, hepatic steatosis might be underdiagnosed in this study, partly due to its retrospective design and the fact that the diagnosis of hepatic steatosis was based on an international classification of diseases-version 10 (ICD-10) code (K76.0), which do not contain NASH, NASH-related fibrosis, NASH-related cirrhosis or hepatocellular carcinoma. In the same study, hepatic steatosis was more common in patients with ACTH-dependent Cushing’s syndrome than in those with ACTH-independent Cushing’s syndrome (27.6% vs. 18.2%, respectively), as well as in men than in women (33.2% vs. 26.0%, respectively) [60], although the latter difference was smaller than that observed in the previously mentioned Chinese study [68]. In a third Chinese study, hepatic steatosis on ultrasonography was present in 33.8% of patients with Cushing’s syndrome [71]. Interestingly, MASLD was also common in children with iatrogenic Cushing’s syndrome; specifically, MASLD was observed in almost half (5 of 11) children on treatment with oral or topical GC [72]. Furthermore, MASLD was observed in this study as early as 2.2 months of life [72].

The above considering, the rates of hepatic steatosis in patients with Cushing’s syndrome (Table 1) appear to be similar or possibly a little higher than those observed in the general population, being around 25–30% [2, 73]. At first glance, this seems to be an apparent paradox, considering the higher rates of abdominal obesity and T2DM observed in patients with Cushing’s syndrome. For example, the global prevalence of NAFLD/MASLD in people with T2DM is 55.5% [74], so only because of the higher rates of T2DM in patients with Cushing’s syndrome, the prevalence of MASLD would be expected to be higher. However, this may be partly attributed to the relatively young age of patients with Cushing’s syndrome included in the above studies and the underestimation of hepatic steatosis with the use of ICD-10 codes or ultrasonography, owing to the relatively low sensitivity of ultrasonography for the detection of mild-to-moderate steatosis, especially in case of obesity, as observed in patients with Cushing’s syndrome [51]. Furthermore, the lower rates of hepatic steatosis than those expected may be attributed to the retrospective design of some studies mentioned above, i.e., they were not explicitly designed for this specific aim. Furthermore, it is not always clear whether the presence of hepatic steatosis was evaluated pre-treatment in all studies, i.e., in treatment naïve patients with Cushing’s syndrome. In some studies, for example, the German one [70], it is clear that patients with Cushing’s syndrome were at various stages of treatment or even on hydrocortisone, being a substitution therapy for adrenal insufficiency after treatment, e.g., the surgery of an adrenal cortisol-secreting adrenal tumor; however, this information is not provided in most of the published studies. It is reasonable to hypothesize that any pharmacological or surgical management of Cushing’s syndrome that affects GC levels may also impact the rates of MASLD in these patients. Some authors have also hypothesized possible anti-inflammatory effects of GC on the liver in patients with Cushing’s syndrome, which may partly counteract the adverse effects of GC on abdominal obesity, IR, T2DM, hypertension and dyslipidemia, which unfavorably affect MASLD [75]; however, this hypothesis remains to be further validated.

**Table 1:** Rates of MASLD in studies with patients with Cushing’s syndrome

**First author (year) country [reference]**^a^	**Patients with Cushing’s syndrome (total; women [N])**	**Age (years)**^b^	**BMI (kg/m**^**2**^**)**^b^	**Methods** **for diagnosing hepatic steatosis**	**Rate of patients with hepatic steatosis (%)**	**Additional information**
Rockall (2003) UK [67]	50; 42	46.6 (14–79)^c^	Women: 29.3 ± 7.2 Men: 25.7 ± 3.3	CT	20.0%	Cross-sectional study; adolescent and adult patients with newly diagnosed Cushing’s syndrome
Liu (2015) China [68]	73; 60	Women: 33.5 (16–62)^c^ Men: 30.0 (14–64)^c^	Women: 24.0 (17.1–34.4)^c^ Men: 27.2 (20.8–35.8)^c^	US	Men: 61.5% Women: 28.6%	Retrospective, cross-sectional study; adult patients with Cushing’s syndrome
Auer (2016) Germany [70]	33; 32	50.3 ± 13.4	26.6 ± 1.0^d^	FLI	30.3%	Case-control study; adult patients with biochemically controlled Cushing’s disease
Zhou (2019) China [60]	1652; 1289	38.1 ± 13.6	NA	ICD-10 code	24.8%	Retrospective, registry-based cross-sectional study; adult patients with Cushing’s syndrome
Güven (2020) Turkey [72]	14; 9	1.76 (0.19–11.9)^c^	24.3 ± 7.8	US (performed in 11 of 14 patients)	45.5%	Retrospective case series; children and adolescents with iatrogenic Cushing’s syndrome
Chen (2023) China [71]	290; 227	38.5 ± 12.8	25.8 ± 4.5	US	33.8%	Retrospective, cross-sectional study; adult patients with active Cushing’s syndrome

*BMI* body mass index, *CT* computerized tomography, *FLI* fatty liver index, *ICD-10* international classification of diseases version 10, *N* number, *NA* not available, *US* ultrasonography

^a^References are sorted according to the publication year

^b^Data presented as mean ± standard deviation, except if it is differently indicated

^c^Median (range)

^d^Mean ± standard error

It should be highlighted that there is no study to date with histologically proven MASLD in patients with active Cushing’s syndrome. The histologic rates of MASH, advanced fibrosis or cirrhosis in patients with Cushing’s syndrome would have been important since they may dictate the outcomes of the disease more strongly than isolated hepatic steatosis [2].

There are also some relevant clinical studies examining the association between the presence and severity of MASLD and subclinical hypercortisolism (albeit not in patients with active Cushing’s syndrome). In a cross-sectional study of 50 patients with biopsy-confirmed MASLD and 40 control subjects who were matched for age, sex and body mass index, Targher et al. showed that overweight patients with biopsy-proven MASLD had a subclinical, chronic HPA axis activation (as reflected by the increased, albeit still normal, 24-h urinary free cortisol values and the blunted suppression of cortisol levels by 1.0 mg-dexamethasone test with normal serum corticosteroid-binding globulin concentrations) that was significantly correlated with the histopathological severity of MASLD (that is, necro-inflammatory grade and fibrosis stage) independently of age, sex, adiposity measures, hypertension, and IR [76]. A significant association between a subtle, chronic overactivity of the HPA axis and MASLD on ultrasonography was also confirmed by the same group of investigators in a previous cross-sectional study of diet-controlled overweight patients with known T2DM [77]. Westerbacka et al. reported that obese men with higher liver fat content (assessed by magnetic resonance imaging spectroscopy) had a selective increase in 24-h urinary excretion of 5-β-reduced cortisol metabolites and a lower ratio of urinary cortisol/cortisone metabolites than their counterparts with lower liver fat content [78]. In line with these findings supporting a subtle HPA axis dysfunction in MASLD, other authors examined the impact of fat distribution, as estimated by several anthropometric parameters, on the severity of liver histology in 123 patients with biopsy-proven MASLD/MASH [79]. They found that dorsocervical lipohypertrophy (also known as “buffalo” hump), which is commonly seen in Cushing's syndrome [80], was the single strongest contributor (rather than body mass index and waist circumference) to the variability in the severity of liver histology in MASLD/MASH [79].

## Possible Treatment Considerations

Since GC have been physiologically associated with multiple actions in diverse organs and systems, their pharmaceutical use is so extensive that iatrogenic hypercortisolism is very common in clinical practice [72, 81]. Therefore, the first consideration to mitigate the long-term adverse effects of GC on the liver and other organs and tissues is their prudent use; indeed, GC should be used only when they are necessary, i.e., when there are no alternative medications tailored for each disease, and at an individual basis, at the minimum daily dose that could control the disease. In addition, when possible, taking a single GC dose in the morning suppresses the HPA axis less and should, therefore, be preferred if this therapeutic scheme effectively controls the disease [81].

Another relevant topic is the pharmacologic inhibition of 11β-HSD1. In line with the above-mentioned association between 11β-HSD1 activity and hepatic steatosis [39, 40] and possibly hepatic inflammation and fibrosis [52, 55], the administration of a selective inhibitor of 11β-HSD1, the 3-(1-adamantyl)-6,7,8,9-tetrahydro-5H-[1,2,4]triazolo[4,3-a]azepine, decreased body weight, and improved lipid profile, fasting glucose and IR in mouse models of diet-induced obesity and diabetes [82]. Similarly, another 11β-HSD1 inhibitor (J2H-1702) significantly improved hepatic steatosis and, most importantly, hepatic fibrosis in diet- and toxicity-induced NASH mouse models [55]. In line with this, other authors recently reported that another 11β-HSD1 inhibitor (H8, a curcumin derivate) alleviated hepatic steatosis and inflammation, mainly by activating the AMPK/Sirtuin-1 signaling pathway, in HFD/streptozotocin-treated rats with MASLD [56].

11β-HSD1 inhibitors have also been used in some phase 1 and 2 randomized controlled trials (RCTs) of patients with MASLD (Table 2) or populations with relevant diseases, including T2DM and obesity. In a double-blind placebo-controlled RCT of 302 patients with inadequately controlled T2DM on metformin monotherapy, a 12-week treatment with an 11β-HSD1 inhibitor (INCB13739) resulted in significant reduction in body weight, hemoglobin A1c, fasting glucose and IR compared with placebo. Moreover, this 11β-HSD1 inhibitor also improved lipid profile in the subgroup of participants with dyslipidemia [83]. However, other authors did not show any significant improvement in body weight and IR in patients with T2DM treated with various 11β-HSD1 inhibitors [84, 85]. Regarding patients with MASLD/NAFLD, two relevant RCTs are summarized in Table 2. In a phase 1b multicenter RCT, treatment with RO5093151 for 12 weeks resulted in improved hepatic steatosis and a greater resolution of MASLD in patients randomly assigned to RO5093151 than placebo [86••]. Liver function tests, body weight, VAT and SAT also decreased after treatment with RO5093151. However, nervous system disorders were more commonly observed in the active drug arm than in the placebo arm (23% vs. 5%), which did not lead to withdrawal from the study. Other potential adverse effects were similar between the two patient groups [86••]. In a phase 2b RCT, treatment with another 11β-HSD1 inhibitor (AZD4017) for 12 weeks did not significantly improve hepatic steatosis, liver function tests, body weight and IR compared with placebo in the sum of participants [87••]. However, treatment with AZD4017 significantly improved hepatic steatosis in the subgroup of participants with MASH and T2DM [87••], thus suggesting a better drug response in participants with more severe disease. It should be noted that AZD4017 blocked the conversion of ^13^C cortisone to ^13^C cortisol in the liver of all participants receiving the drug. The most commonly reported adverse effects of AZD4017 were gastrointestinal ones (diarrhea, softer tools, and stomachache) and headache [87••]. More recently, J2H-1702 was evaluated in a phase 1 RCT (involving 50 healthy Korean men) [88••], after having provided favorable results in preclinical models, as mentioned above [55]. The authors of this phase 1 RCT showed that after a single dose of 300 mg, the maximal inhibitory effect of J2H-1702 was reached after 12–24 h, its inhibitory effect was dose-dependent and was maintained for one day [88••]. Diarrhea and dizziness were the most commonly reported adverse effects, which were mild and resolved spontaneously [88••]. Based on these findings, a multicenter, phase 2a RCT with J2H-1702 in patients with NASH/MASH is ongoing (Clinical Research Information Service ID: KCT0007904). Although the results of the trial are highly expected, the use of novel hepatic-selective 11β-HSD1 inhibitors will be investigated soon to reduce the potential long-term adverse effects of these drugs on other extrahepatic tissues.

**Table 2:** Randomized controlled trials of 11β-HSD1 inhibitors in adult patients with MASLD

**First author (year)[reference]**^a^	**Study design**	**Patients (N)**	**11β-HSD1 inhibitor; trial duration**	**Main findings**
Stefan (2014) [86••]	Multicenter, double-blind RCT (phase 1b); MASLD was diagnosed by MRS	82 MASLD patients (41 on active arm; 41 on placebo arm)	RO5093151 (200 mg, twice/day); 12 weeks	Liver fat content was improved after treatment with 11β-HSD1 inhibitor compared to placebo
Yadav (2022) [87••]	Two-center, double-blind RCT (phase 2b); MASLD was diagnosed by MRI-PDFF or liver biopsy	93 MASLD patients (46 on active arm; 47 on placebo arm)	AZD4017 (400 mg, twice/day); 12 weeks	Changes in liver fat content were comparable between the two treatment arms. However, in the subgroup of patients with MASH and T2DM, liver fat content was improved after treatment with 11β-HSD1 inhibitor compared to placebo

*11β-HSD1* 11beta-hydroxysteroid dehydrogenase type 1, *MASLD* metabolic dysfunction-associated steatotic liver disease, *MRI-PDFF* magnetic resonance imaging-proton density fat fraction, *MRS* magnetic resonance spectroscopy, *N* number, *MASH* metabolic dysfunction-associated steatohepatitis, *RCT* randomized controlled trial, *T2DM* type 2 diabetes mellitus

^a^References are sorted according to the publication year

Some reports have also shown that mifepristone, a GR competitive antagonist, might benefit MASLD. Weight loss and decreased serum aminotransferase levels were reported in a female patient with Cushing’s syndrome and biopsy-proven NASH after 20 weeks of mifepristone treatment [89]. Besides its beneficial effect on liver function tests, long-term mifepristone treatment also reduced hepatic fat content (imaging) in two women with Cushing’s syndrome [90•]. A single mifepristone dose (400 mg) with metyrapone (1 g), the latter being an inhibitor of cortisol biosynthesis, was also shown to improve fasting glucose, IR and hepatic glucose production in patients with T2DM, regardless of the presence or absence of MASLD [91]. Nevertheless, despite these encouraging results, we could not support the investigation of mifepristone in future clinical trials of MASLD patients without active Cushing’s syndrome due to its long-term adverse effects, including symptoms of cortisol withdrawal, hypokalemia and changes in thyroid function parameters, effects owing to its antiprogesterone activity, and rash [92]. However, the observed beneficial effects of mifepristone are another piece of evidence supporting a link between GC and MASLD. Furthermore, although the level of evidence is low (case reports), mifepristone may be used, after careful clinical judgment and under close patient monitoring [92], in patients with active Cushing’s syndrome and MASLD who cannot be subjected to pituitary or adrenal surgery or who deny surgery.

That said, it should also be underlined that not all drugs administered for treating chronic hypercortisolism have beneficial effects on the liver. A classic example is ketoconazole, a drug against fungal infections used in Cushing’s syndrome, since it inhibits cortisol biosynthesis [93]. Ketoconazole can adversely affect liver function tests, so a routine assessment of liver function tests is strongly recommended [93]. In this regard, ketoconazole should be avoided or used carefully in patients with active Cushing’s syndrome and MASLD, at least until the publication of relevant data. Other medications under evaluation for treating chronic hypercortisolism may also adversely affect MASLD. For example, epidermal growth factor receptor (EGFR) inhibitors are under investigation for treating Cushing’s disease since EGFR receptors are highly expressed in human pituitary corticotroph adenomas [93]. However, the loss of EGFR expression seems to contribute to the pathogenesis of MASLD, at least in preclinical animal models [94]. This may be considered in future clinical trials of patients with Cushing’s disease.

Another relevant topic is the use of 5α-reductase inhibitors, primarily used in patients with benign prostate hyperplasia. Indeed, while 5α-reductase inhibitors decrease plasma dihydrotestosterone concentrations, which is required in patients with benign prostate hyperplasia, these inhibitors may also increase GC concentrations, which represents an adverse drug effect [95]. Regarding MASLD, dutasteride (i.e., a dual 5α- and 5β-reductase inhibitor), but not finasteride (a selective 5β-reductase inhibitor), increased hepatic steatosis after 3-week treatment in 12 healthy men [96]. Although further studies are needed, other authors have also highlighted the potential adverse effects of 5α-reductase inhibitors on MASLD and other metabolic and non-metabolic diseases, including T2DM and chronic kidney disease [97].

## Closing Remarks

MASLD is a highly prevalent metabolic liver disease worldwide without any approved pharmaceutical treatment [2]. The pathogenesis of MASLD is complex and multifactorial [6]. In this regard, GC, commonly used for many inflammatory and autoimmune diseases, may contribute to the development and progression of MASLD, acting in parallel with other pathogenic contributors [8]. Regarding the pathophysiology of GC-induced MASLD, GC are closely implicated in regulating carbohydrate, protein and lipid metabolism, as well as energy homeostasis [13, 14]. By acting mainly through their specific intracellular GR, GC may induce hyperglycemia and hyperinsulinemia, thus increasing systemic and hepatic IR [22], a hallmark of the pathogenesis of MASLD [10]. Furthermore, GC may increase adipose tissue lipolysis [32], increase hepatic *de novo* lipogenesis and decrease hepatic fatty acid β-oxidation, thus promoting hepatic steatosis [37]. Apart from hepatic steatosis, preclinical evidence also indicates that GC may adversely affect hepatic inflammation and fibrosis [52, 55]. 11β-HSD1, a regulatory enzyme converting the inactive cortisone to active cortisol [38], seems to play a key role in the link between GC and MASLD since it was shown to enhance the GC action in the liver. Notably, the pharmacologic inhibition of 11β-HSD1 has provided some favorable results for MASLD, both in preclinical studies [55, 56, 82] and early MASLD clinical trials [86••, 87••].

A clinical paradigm of the long-term effects of GC excess is Cushing’s syndrome [15], characterized by abdominal obesity, dyslipidemia, hypertension and T2DM [58], which are all related to the pathophysiology of MASLD [1••, 5, 59]. However, further studies, preferably with liver histological confirmation, are required to evaluate the rates of MASH and liver fibrosis, specifically in patients with Cushing’s syndrome. In addition, based on the available evidence discussed above, we believe additional studies are also needed to better understand whether targeting 11β-HSD1 might be a promising approach for appropriate combination therapy of MASLD.

## Data Availability

No datasets were generated or analysed during the current study.
